# Modulation of Efficient Diiodo-BODIPY *in vitro* Phototoxicity to Cancer Cells by Carbon Nano-Onions

**DOI:** 10.3389/fchem.2020.573211

**Published:** 2020-10-06

**Authors:** Juergen Bartelmess, Gesmi Milcovich, Viviana Maffeis, Marta d'Amora, Sine Mandrup Bertozzi, Silvia Giordani

**Affiliations:** ^1^Nano Carbon Materials, Istituto Italiano di Tecnologia (IIT), Genoa, Italy; ^2^School of Chemical Sciences, Dublin City University (DCU), Dublin, Ireland

**Keywords:** carbon nano-onions, photodynamic therapy, reactive oxygen species, cancer treatments, photosensitizer

## Abstract

Photodynamic therapy is currently one of the most promising approaches for targeted cancer treatment. It is based on responses of vital physiological signals, namely, reactive oxygen species (ROS), which are associated with diseased condition development, such as tumors. This study presents the synthesis, incorporation, and application of a diiodo-BODIPY–based photosensitizer, based on a non-covalent functionalization of carbon nano-onions (CNOs). *In vitro* assays demonstrate that HeLa cells internalize the diiodo-BODIPY molecules and their CNO nanohybrids. Upon cell internalization and light exposure, the pyrene–diiodo-BODIPY molecules induce an increase of the ROS level of HeLa cells, resulting in remarkable photomediated cytotoxicity and apoptosis. Conversely, when HeLa cells internalize the diiodo-BODIPY/CNO nanohybrids, no significant cytotoxicity or ROS basal level increase can be detected. These results define a first step toward the understanding of carbon nanomaterials that function as molecular shuttles for photodynamic therapeutics, boosting the modulation of the photosensitizer.

## Introduction

Boron complexes of dipyrromethenes, so-called BODIPY dyes, have been established as suitable fluorophores for several applications because of their synthetic flexibility, high photostability, and bright fluorescence (Loudet and Burgess, 2007; Hinkeldey et al., 2008; Ulrich et al., 2008). Based on the multiple chances to synthetically modify the BODIPY fluorophore, the tuning of the excited state properties of BODIPY dyes toward efficient triplet photosensitizers can be realized by the introduction of diiodo-substituents on the BODIPY core (Yogo et al., 2005; Loudet and Burgess, 2007). In the biomedical field, the application of BODIPY dyes as singlet oxygen sensitizers for photodynamic therapy (PDT) has emerged as a significant therapeutic approach for cancer treatment (Awuah and You, 2012; Kamkaew and Burgess, 2013; Kamkaew et al., 2013). Furthermore, the photodynamic inactivation of microbes, fungi, and viruses by diiodo-substituted BODIPY derivatives was demonstrated and found to be photosensitizer dose and light source exposure dependent; their precise modulation can trigger the eradication of the bacterial strains (Caruso et al., 2012; Carpenter et al., 2015). Most modern PDT applications involve three key components: a photosensitizer, a light source (i.e., a laser or LED lamps), and oxygen. As a consequence, the activation of cytotoxic cascade signals takes place, such as reactive oxygen species (ROS), including superoxide. Upon irradiation, the excited photosensitizer transfers energy to triplet oxygen, resulting in the generation of ROS, which can be exploited to destroy cancer cells. Nevertheless, ROS overexpression is connected to oxidative stress, which can turn into a powerful asset to face tumor conditions development (Liou and Storz, 2010; Lee et al., 2013). ROS are intermediate metabolism products; they induce a disruptive effect on the cell membrane, impairing sulfhydryl bonds and stimulating lipid peroxidation (Wiseman and Halliwell, 1996). In physiological conditions, healthy tissues are characterized by low levels of reactive radical species (Milcovich et al., 2017). However, in pathological events, such as tumors or inflammatory diseases, the cytotoxic cascade is activated because of an overtaking in the safe ROS level threshold. Therefore, cancer cells require a remarkably lower ROS increase to obtain cytotoxicity, compared to healthy cells. This feature renders PDT an outstanding approach toward the study and development of precisely targeted tumor treatments. A complete understanding of the ROS-triggered apoptotic mechanisms of diiodo-substituted BODIPY derivatives is a crucial milestone (Portney and Ozkan, 2006; Zhu et al., 2014; Raju et al., 2015). In this context, the study of their coupling with carbon nanomaterials (CNMs) plays a vital role in thoroughly investigating the modulation of such a cascade (Kostarelos et al., 2009; Fabbro et al., 2012). The non-covalent approach for CNM functionalization is a core method; (Singh et al., 2009) pyrene moieties are exceptionally well-established for decorating CNMs with functional groups through non-covalent functionalization (Zhao and Stoddart, 2009). We recently reported the non-covalent functionalization of carbon nano-onions (CNOs; a multilayer fullerene-like graphitic CNM) with highly fluorescent pyrene–BODIPY dyads and their application for biomedical imaging, with promising results (Bartelmess et al., 2015). These findings comply with other recent works, where CNOs exhibited a prompt and effective uptake by different cell lines, (Frasconi et al., 2015; Lettieri et al., 2017a,b; d'Amora et al., 2019) revealing high stability, low toxicity, and high biocompatibility in zebrafish during their development (d'Amora et al., 2016, 2017).

**Scheme 1:**
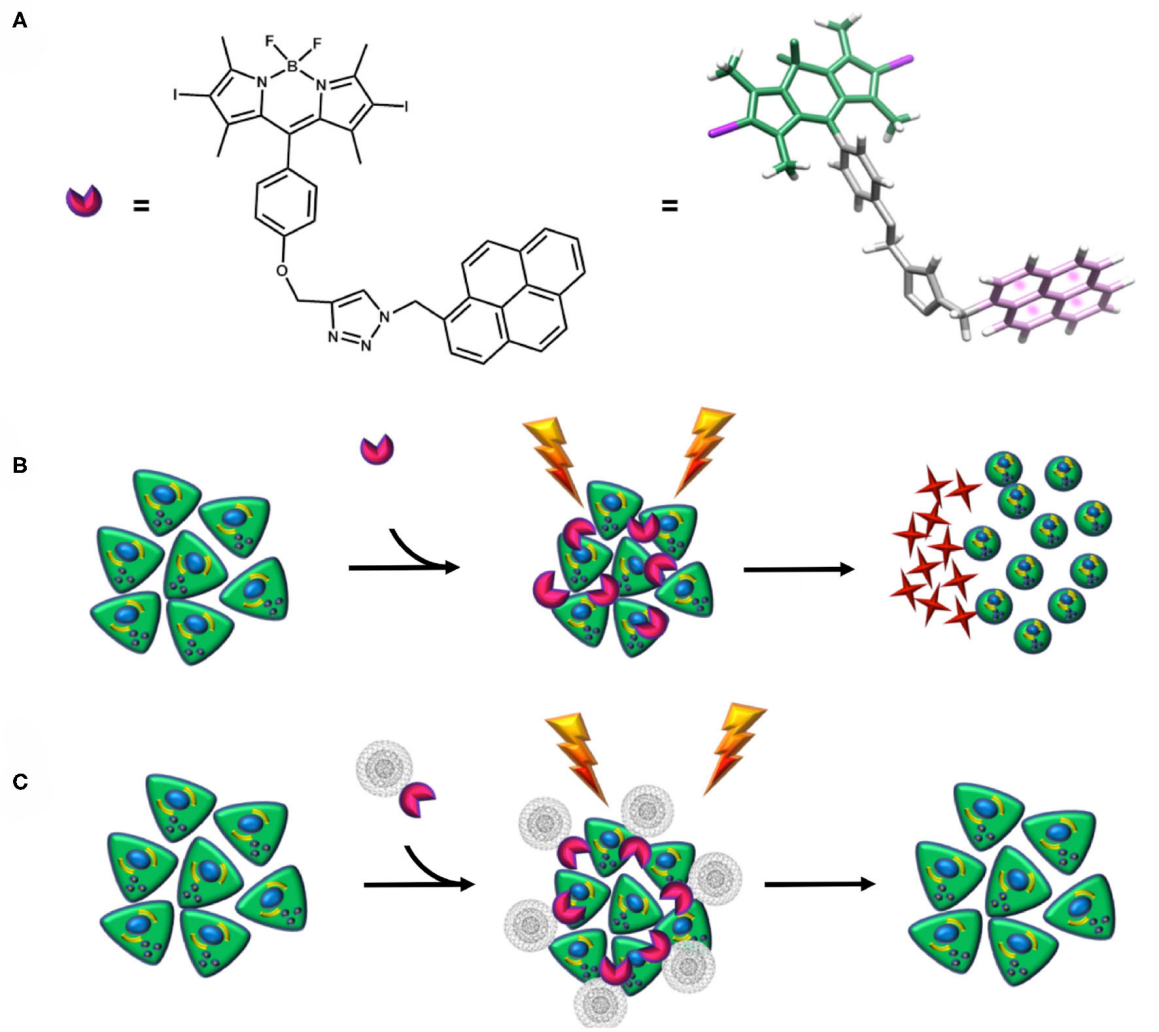
**(A)** Structure of the photosensitizer. **(B,C)** Schematic representation of the diiodo-BODIPY–based photosensitizer apoptotic mechanism and its non-covalent functionalization with CNOs. When the photosensitizer is irradiated, a ROS-mediated cascade reaction takes place, leading to a microenvironmental inflammatory event, cytotoxicity, and further apoptosis **(B)**. Conversely, when the photosensitizer is coupled with CNOs, no cytotoxicity is detected **(C)**.

The aim of this work relies on the synthesis of a nanohybrid composed of a novel pyrene–diiodo-BODIPY dyad, acting as an efficient photosensitizer, and CNOs. Two different CNOs are tested: pristine CNOs (p-CNOs) and benzoic acid–functionalized CNOs (benz-CNOs), the latter providing an enhanced water dispersability. The photosensitizer and the nanohybrids are characterized by optical spectroscopy. Moreover, the photodynamic efficiency of the dye and the corresponding nanohybrids, and their cellular uptake by HeLa cells, are investigated (Scheme 1).

## Experimental

### Materials

All reagents and solvents were purchased from Sigma–Aldrich in high purity and used as received. All reactions and measurements were carried out under ambient conditions, unless otherwise specified.

### Synthesis of BODIPY–Pyrene Derivative 3

Propargylated diiodo-BODIPY derivative **1** (Erbas et al., 2009) and 1-(azidomethyl)pyrene **2** (Bartels et al., 2009) were synthesized following published procedures.

**Scheme 2:**
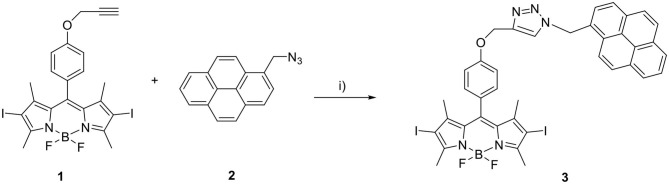
Synthesis of BODIPY-pyrene derivative **3**. (i) CuI, dry Et_3_N, dry THF, N_2_, 20 h, RT, dark.

**1** (50 mg, 0.08 mmol), **2** (41.7 mg, 0.16 mmol), and copper(I) iodide (16 mg, 0.16 mmol) were dissolved in 9 mL of dry THF under inert nitrogen atmosphere on a Schlenk line. Dry Et_3_N (410 μL, 4 mmol) was added, and the reaction mixture was stirred at room temperature (RT) for 20 h in the dark (see Scheme 2).

The reaction mixture was filtered through a pad of silica, eluted with dichloromethane and dried. The crude was purified by two subsequent column chromatographic steps [1: SiO_2_, eluted with hexane: dichloromethane (1:1, vol/vol); 2: SiO_2_, eluted with hexane: acetone (2:1, vol/vol)]. A microcrystalline precipitate crystallized readily from the dark red solution, which was recovered from the 2nd column, upon addition of hexane. Yield: 39.6 mg (0.045 mmol, 56 %). ^1^H nuclear magnetic resonance (NMR) (400 MHz, CDCl_3_):δ:8.14 (m, 9H), 7.38 (s, 1H), 7.01 (d, 4H), 6.31 (s, 2H), 5.15 (s, 2H), 2.63 (s, 6H), 1.33 (s, 6H). HR-MS: calculated mass for C_39_H_31_BF_2_I_2_N_5_O [M+H]^+^: 888.0686; measured: 888.0691 (see Supplementary Material for characterization spectra/data).

### Preparation of 3/CNOs and 3/Benz-CNOs Nanohybrids

**3** (4 mg, 0.006 mmol) and 15 mg of p-CNOs (Palkar et al., 2007) were dispersed in 15 mL of dry dimethylformamide and sonicated in an ultrasonic bath for 30 min. Then, the CNOs were filtered through a nylon filter (0.2 μm) and washed with THF until the filtrate appeared colorless. Finally, the functionalized CNO nanomaterial was dried overnight at RT. Fifteen milligrams of **3/CNOs** hybrid was recovered from the filter as a black, fine powder. The preparation of **3/benz-CNOs** was accomplished in a similar manner.

### Characterization of 3, 3/CNOs, and 3/Benz-CNOs

Absorption spectra were recorded on an Agilent Cary 8454 UV-Vis diode array spectrophotometer. Corresponding fluorescence spectra were taken on a Horiba Jobin Yvon Fluoromax-4 spectrofluorometer in 1.00 × 1.00-cm quartz glass cells. Fluorescence quantum yields were determined by the comparative method of Williams et al. (1983). The integrated fluorescence intensities of a known dye and the tested compound were compared, and fluorescence quantum yields were calculated using the following equation:

$$\begin{array}{l} \Phi_{x}=\left(\Phi_{st}\right)\left(Grad_{x}/Grad_{st}\right)\left(\eta_{x}^{2}/\;\eta_{st}^{2}\right) \end{array}$$

*st* and *x* denote the standard and test, respectively, whereas Φ is the fluorescence quantum yield. *Grad* is the gradient obtained from the plot of integrated fluorescence intensity vs. absorbance of the dye at the excitation wavelength. η Represents the refractive index of the used solvents. The fluorescence quantum yield of **3** was measured relative to *meso*-phenol-1,3,5,7-tetramethyl-BODIPY with Φ_*St*_ = 0.64 in toluene (Lazarides et al., 2012). Singlet oxygen quantum yields were measured and compared to rose Bengal in benzyl alcohol, using 1,3-diphenylisobenzofuran (DPBF) as a singlet oxygen acceptor probe. Additional photobleaching experiments were carried out in benzyl alcohol, comparing the loss of fluorescence of DPBF in the presence of BODIPY **3, 3/CNOs & 3/benz-CNOs** (CNO samples of comparable mass concentrations; 10 μg mL^−1^) at different illumination time points (see Supplementary Material). The accurate mass measurements (HRMS) were performed on a Waters SYNAPT G2 High Resolution Mass Spectrometry instrument equipped with an electrospray ionization interface and coupled to a Waters ACQUITY UPLC. Electrospray ionization in positive mode was applied in the mass scan range 50–1,200 Da. The analysis was conducted on a Waters ACQUITY UPLC BEH C18 column 100 × 2.1-mm ID (particle size 1.7 μm) with an in-line filter. The mobile phase was 0.1% formic acid in water and 0.1% formic acid in acetonitrile. NMR spectroscopy was performed on a Bruker Avance III 400 MHz system (400.13 MHz for ^1^H) in CDCl_3_. NMR spectra has been processed with Mnova.

### Cell Culture

HeLa wild-type cells (derived from a human cervix carcinoma, ATCC® supplied) were cultured in Dulbecco modified Eagle medium (DMEM) (Life Technologies) supplemented with 10% fetal bovine serum (Life Technologies), 2% penicillin streptomycin (PenStrep) (Life Technologies), and 1% glutamine (Life Technologies) at 37°C in a humidified 5% CO_2_ atmosphere.

### Cytotoxicity Assays

For the cytotoxicity experiments, the CNO samples were prepared by suspending 1 mg of sample in 2 mL sterile phosphate-buffered saline (PBS; 0.1 M, pH 7.4), followed by ultrasonication for 20 min, for a mother solution of a 0.5 mg mL^−1^ mass concentration. The CNOs were then dispersed in DMEM, to obtain CNO dispersions at final mass concentrations of 10 and 20 μg mL^−1^. Mother solutions of **3** in dimethyl sulfoxide (DMSO) were also prepared and diluted into DMEM medium with comparable concentrations to the CNO-based samples. The same protocol was applied in order to prepare the **3/CNO** samples: the nanohybrids were obtained by preparing a DMSO mother solution of the same CNO mass concentration. Cells were seeded in 96-well plates at a density of 4 × 10^3^ cells/well and cultured overnight in 150 μL of medium per well. Further on, cells were incubated with the different concentrations of CNO dispersions for 24 h, washed three times with PBS, and covered with DMEM (phenol red–free). The plates were illuminated using a commercially available LED lamp from Philips (11 W, 2,700 K, 1,065 lm). After illumination (0, 5, 15 min), the phenol red–free medium was replaced with regular DMEM cell medium, and the cells were incubated for 24 h. The cellular viability of the HeLa cells was evaluated utilizing the PrestoBlue® cell viability assay (Life Technologies). Assays were performed following a procedure previously reported (Jokerst et al., 2012), based on the measurement of the absorbance at 570 nm, on a microplate reader. According to the manufacturer's instruction, absorbance measurements at 600 nm were subtracted to the 570-nm acquisitions. Each measurement was normalized with the average signal of control experiment (untreated cells), and cell viability was expressed in terms of metabolic activity as the mean ± SD.

### Cellular Uptake and Oxidative Stress Qualitative Assays

HeLa cells were plated in 4-well Nunc™ chamber slides (–Aldrich) at a density of 5 × 10^3^ cells/well and cultured overnight in the maintenance medium at 37°C in a 5% CO_2_ humidified incubator. The day after, HeLa cells were treated with a mass concentration of 10 μg mL^−1^ of **3/CNO** (CNOs initially dispersed in PBS, DMEM, phenol red–free solution, 2% DMSO) for 24 h. The samples were then washed three times in PBS (0.1 M, pH 7.4) and incubated with 10 μM Hoechst 33342 (Sigma–Aldrich) diluted in DMEM (1:1,000) for 15 min at 37°C, to label the nuclei. Cells were rinsed three times in PBS (0.1 M, pH 7.4). Finally, to analyze the intracellular localization of **3/CNO**, the cells were visualized with a laser scanning confocal microscope, equipped with a resonant scanner (Nikon A1R). Excitation of the **3** was performed at 488 nm, and the emission was acquired in the spectral window between 520 and 580 nm, whereas the Hoechst 33342 was excited at 405 nm, with images acquired in the emission range of 415–480 nm. Cell oxidative damage was revealed by staining for 30 min with 5 μM Cell ROX® Deep Red reagent (Invitrogen™). Cell ROX® was excited at 644 nm, and emission was detected in 655 nm.

### ROS/Superoxide Quantification Assay

Cells were seeded in 96-well, black-wall, clear-bottom plates, at a density of 2 × 10^3^ cells/well and cultured overnight in 150 μL of medium per well. The **3**, **CNOs** & **3/CNOs** samples were prepared as previously described for cytotoxicity assays (DMEM with 2% DMSO was used). Cells were incubated with the different concentrations of **3**, **CNOs** and **3/CNOs** solutions for 24 h and washed three times with PBS. DMEM medium (phenol red–free) was then added, and the plates were illuminated for 5 and 15 min. Cells were then treated with the same PBS washing protocol, eventually replacing PBS with DMEM medium (phenol red–free). Positive control (pyocyanine, ROS inducer) and negative control [*N*-acetyl-l-cysteine (NAC), ROS inhibitor] were added to the selected wells and incubated for 30 min, at 37°C in a 5% CO_2_ humidified incubator, according to the kit instructions. The ROS detection mix (Abcam® cellular ROS/superoxide assay detection kit), suspended in phenol red–free DMEM, was then added and incubated for 60 min. A fluorescence microplate reader was used, calibrated on standard orange (Ex/Em = 550/620 nm) filter set for superoxide species.

### Statistical Analysis

All statistical analysis was performed using Minitab Express, version 1.5.0. Data were compared using one-way analysis of variance, with Dunnett test for comparison procedures. Values are expressed as the mean ± standard deviation and are considered as significantly different with a *p* < 0.05.

## Results and Discussion

The synthesis of the pyrene–diiodo-BODIPY dyad **3** was accomplished by a *Huisgen*-type “click” reaction of a propargylated diiodo-BODIPY (Erbas et al., 2009) and 1-(azidomethyl)pyrene (Bartels et al., 2009) (Scheme 2). Despite previously reported non-iodated pyrene–BODIPY dyad, (Bartelmess et al., 2015) the “click” reaction failed in the presence of catalytic amounts of CuI and sodium ascorbate. However, an excess of CuI and triethylamine, without ascorbate, led to the desired compound **3**. Following this, **3** was immobilized on the surface of pristine, nanodiamond-derived CNOs (Palkar et al., 2007) by ultrasonication, subsequent filtration, and removal of excess dye by washing; as per our established protocol, (Bartelmess et al., 2015) leading to **3/CNOs**. Additional experiments were carried out with benzoic acid functionalized CNOs (**3/benz-CNOs**), in order to study the influence of different CNO surface modifications. **Benz-CNO**s were prepared based on our previous reports (Frasconi et al., 2015; Lettieri et al., 2017b). The solubility of **3** proved to be good only in non-polar solvents. **3** was characterized by absorption and fluorescence spectroscopy (Table 1). The absorption maximum of **3** in DMSO and chloroform was located at 536 nm with molar extinction coefficients of 79.9 × 10^3^ and 89.6 × 10^3^ M^−1^ cm^−1^, respectively. In toluene, the absorption maximum was at 537 nm with a molar extinction coefficient of 83.5 × 10^3^ M^−1^ cm^−1^.

**Table 1:** Photophysical properties of **3**.

**Solvent**	****λ**_Abs_** **max (nm)**	**ε(M^**−1**^ cm^**−1**^)**	**λ_Em_** **max (nm)**	**Stoke shift (nm)**	****Φ_F_****
Toluene	537	83.5 × 10^3^	554	17	0.03
Chloroform	536	89.6 × 10^3^	552	16	0.03
DMSO	536	79.9 × 10^3^	555	19	0.01

**Figure 1:**
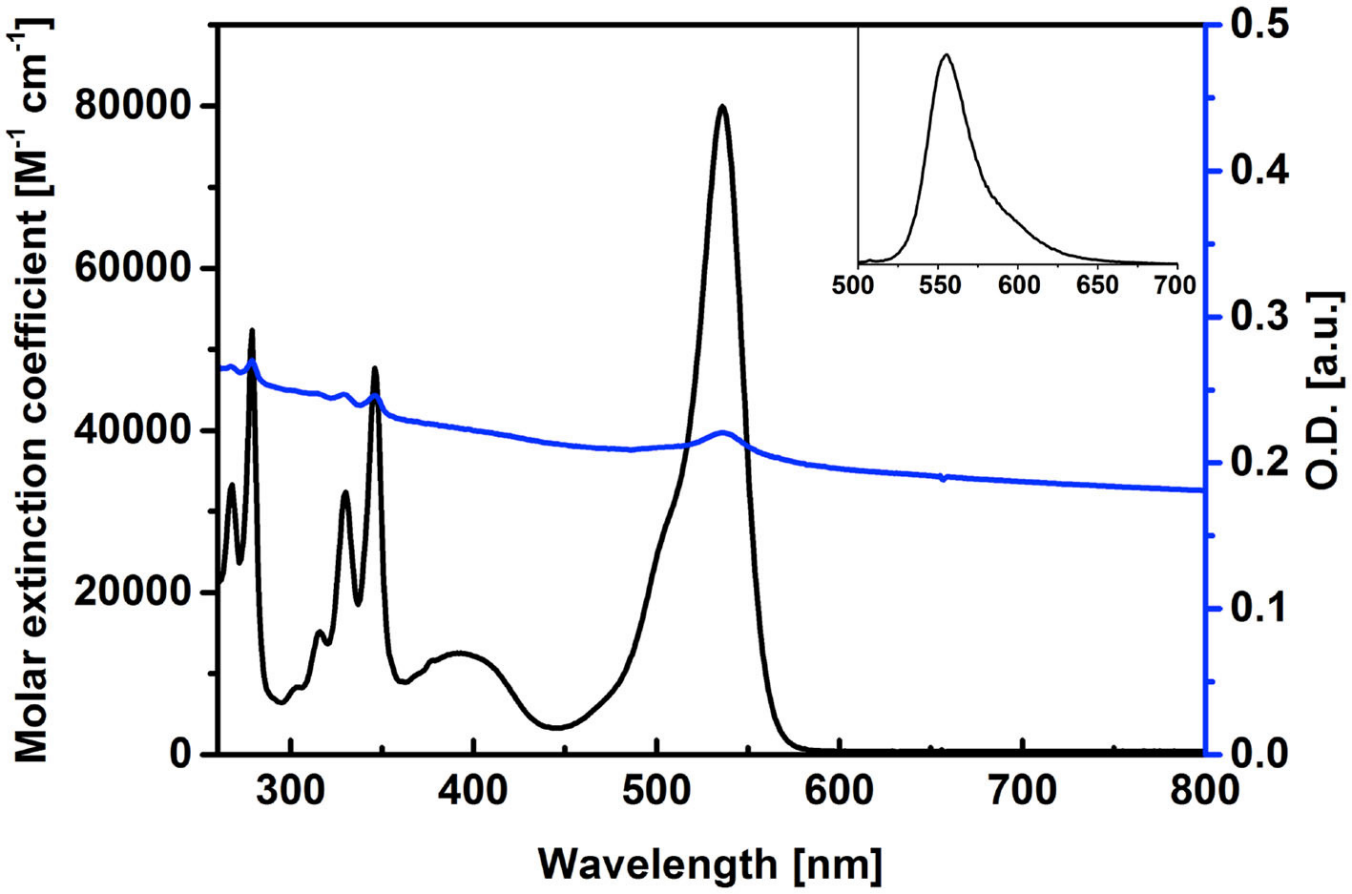
Absorption spectra of **3** (black) and of a dispersion of **3/CNOs** (blue, 10 μg mL^−1^) in DMSO, reporting the molar extinction coefficient of **3** and the loading of the CNOs with dyad **3**. Inset: fluorescence spectrum of **3**, upon excitation at 490 nm.

Pyrene-centered absorption bands were observed at 268, 279, 316, 330, and 346 nm, with an additional broad, BODIPY-based absorption band located in the area around 400 nm in DMSO (Figure 1). The fluorescence quantum yield of **3** was low, due to the heavy-atom effect of the iodosubstituents on the BODIPY core structure, with values between 1 and 3% and emission maxima between 552 and 555 nm (Figure 1, inset). The singlet oxygen quantum yield of **3** was found to be 0.37, compared to rose Bengal in benzyl alcohol. The photophysical characterization of **3/CNOs** clearly revealed the loading of the CNO nanoparticles with **3**. A dispersion of **3/CNOs** in DMSO showed the plasmonic absorption and scattering of the CNOs nanomaterial over the whole spectral area. Furthermore, distinct absorption peaks at 279, 329, 346, and 536 nm are related to the presence of **3** in an estimated concentration of 2.3 × 10^−7^ M, for a **3/CNO** dispersion with a mass concentration of 10 μg mL^−1^ (Figure 1). A weak fluorescence signal related to the BODIPY fluorophore was observed at 555 nm, following excitation at 490 nm. The concentration of **3** in a 10 μg mL^−1^ DMSO dispersion of **3/benz-CNOs** was determined to be about 7.7 × 10^−8^ M (1/3 compared to the **3/CNO** dispersion). Thus, the observed degree of non-covalent CNO functionalization is closely related to previously reported CNO functionalization with highly fluorescent pyrene–BODIPY dyads (Bartelmess et al., 2015).

**Figure 2:**
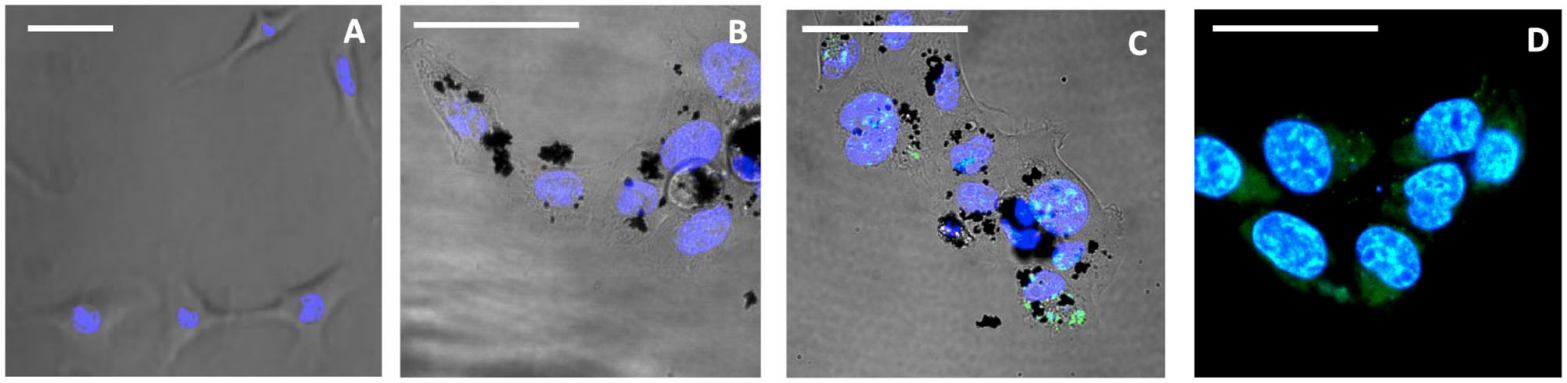
Confocal images of live HeLa cells, not exposed to illumination. **(A)** Untreated control, **(B)** treated with CNOs, incubated for 24 h, **(C)** incubated for 24 h with 3/CNOs at a mass concentration of 10 μg mL^−1^, **(D)** incubated with 3, at the corresponding concentration of 2.3 × 10^−7^ M. Nuclei were stained with Hoechst 33342 (blue), whereas green refers to the I_2_-BODIPY signal. Scale bars **(A–D)** = 50 μm.

The cellular uptake and cytotoxicity of **3/CNOs** and **3/benz-CNO** were investigated to evaluate the activity of nanohybrids and determine the apoptotic mechanisms of the photosensitizer **3**, in the presence and absence of CNOs. For the uptake studies, HeLa cells were incubated with a dispersion of **3/CNOs** at a mass concentration of 10 μg mL^−1^ in DMEM medium (Figure 2). Despite the relatively low fluorescence quantum yield of **3**, an efficient uptake of **3/CNOs** into the cancer cells after 24 h of incubation was observed by the presence of a green fluorescence signal of the BODIPY dye. This finding confirms the uptake and intracellular localization of the BODIPY inside HeLa cells.

**Figure 3:**
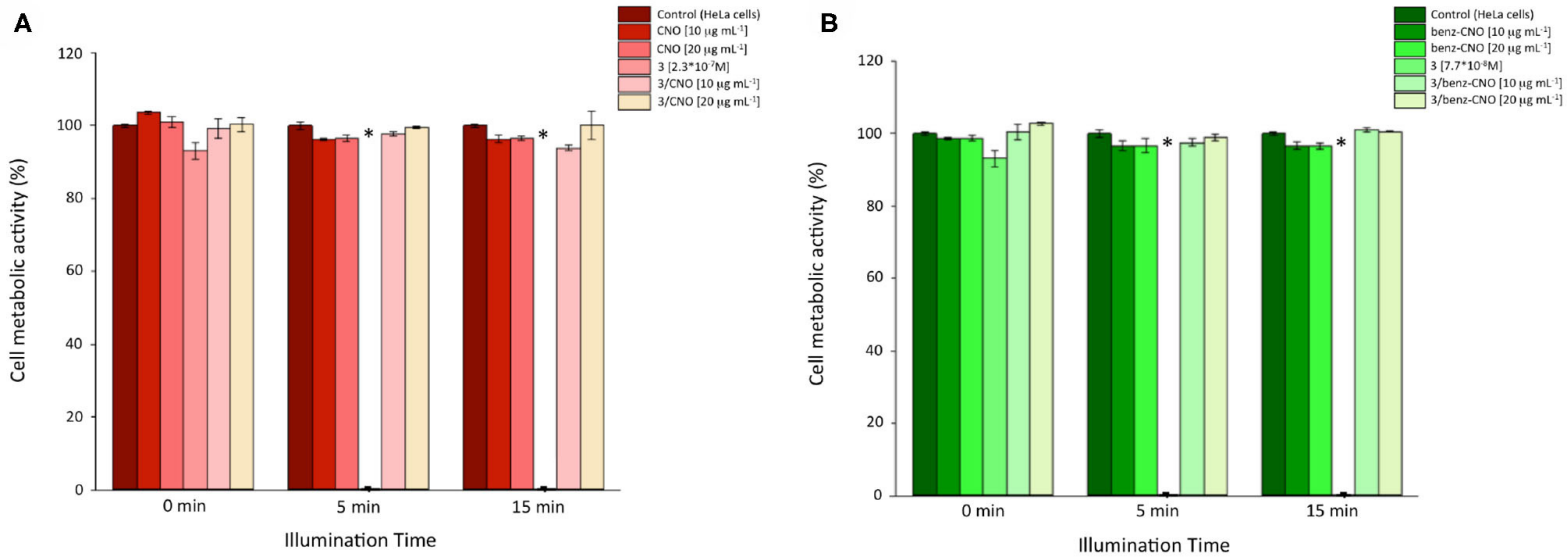
Cellular metabolic activity of HeLa cells incubated with different mass concentrations of **3/CNOs (A)** and **3/**benz-CNOs **(B)**, following illumination at different time points (*n* = 3, *p* < 0.05). *Worldwide acknowledged as to be used to define data which is out of the mean reference range and represents all statistically different data from the reference (controls).

For cytotoxicity studies, HeLa cells were incubated with dispersions of **benz-CNOs** and **3/CNOs** of different mass concentrations (10 and 20 μg mL^−1^) for 24 h. Final working dilutions were obtained by dispersing CNOs from mother solutions in DMSO, in DMEM. Following the incubation, the cells were washed with PBS three times and illuminated with the white LED described in the Experimental section. Illumination exposure time points were set to 5 and 15 min. Non-irradiated experiments were conducted as well (dark control, *T* = 0). Furthermore, a 2.3 × 10^−7^ M solution of **3** was used as a reference, based on the estimated loading of **3** onto the nanohybrids. DMSO was added to all samples to obtain a final DMSO concentration of 2% vol. The time-dependent phototoxicity of the treated cells was quantified using the Presto Blue® assay. Photosensitizer **3** alone revealed impressive cytotoxicity after illumination (Figure 3). After 5 min, no cellular metabolic activity could be detected, while in the dark control, the metabolic activity was not impaired by the exposure to the dye. The coupling of **3** with both **CNOs** and **benz-CNOs** did not induce any illumination time or mass concentration–dependent reduction of HeLa metabolic activity. Therefore, the nanohybrid formation prevents the massive cytotoxicity of the photosensitizer.

The cytotoxicity assays indicated that the presence of CNOs inhibited the photodynamic efficiency of the BODIPY photosensitizer **3**.

Data derived from additional photobleaching experiments supported these results by describing the ability of the photosensitizer **3** to generate ROS (Supplementary Figure 4). The BODIPY dye **3** without CNOs showed high efficiency in ROS generation, indicated by the rapid photobleaching of the ROS sensor molecule DPBF. When **3/CNO** nanohybrids were used, the photobleaching of the DPBF is slowed, but still progresses. These findings suggest that partial removal of the dye from the CNO surface in organic solvents, such as benzyl alcohol (Supplementary Figure 4) or DMSO (in the *in vitro* experiments), could take place. In addition, the lower loading of the **3/benz-CNOs**, compared to the **3/CNO** hybrid, was confirmed by this set of data.

Overall, comparable results were obtained for **3/benz-CNOs** (see Supplementary Material). Lower concentrations of **3** still confirmed a sharp photodynamic-triggered reduction of the cellular metabolic activity, whereas when immobilized onto the CNOs, **3** did not reveal significant cytotoxicity.

**Figure 4:**
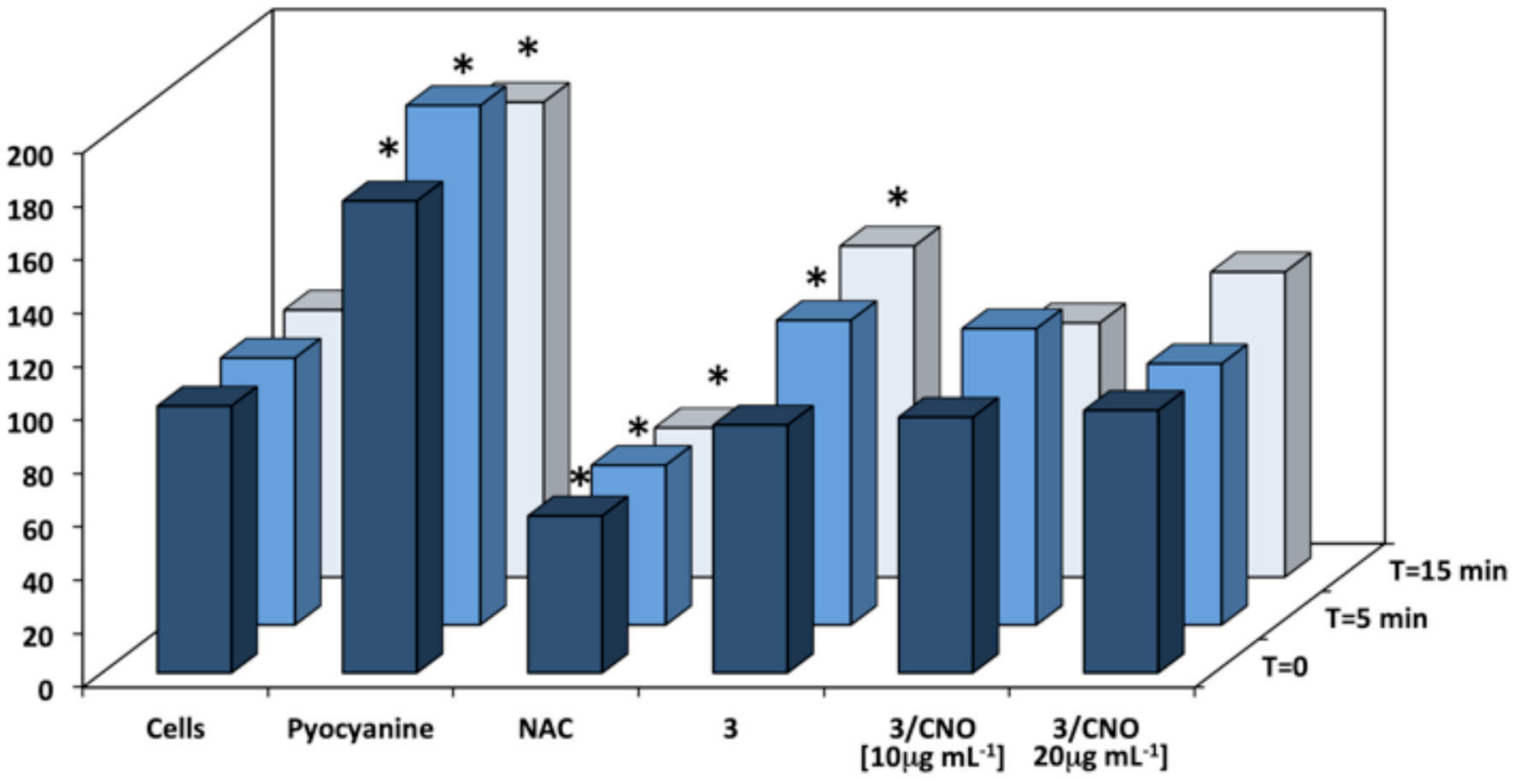
Quantification of superoxide levels in HeLa cells incubated with different mass concentrations of **3** or **3/CNOs**, following illumination at different time points (*n* = 3, *p* < 0.05). Control (cells), positive control (pyocyanine, ROS inducer), and negative control [N-acetyl-l-cysteine (NAC)] are also tested. *Worldwide acknowledged as to be used to define data which is out of the mean reference range and represents all statistically different data from the reference (controls).

A quantification of *in vitro* ROS and superoxide species was studied, to determine whether the cytotoxic and apoptotic effects of the photosensitizer were due to the activation of the ROS cascade mechanism. Basal superoxide level, positive control (pyocyanine, ROS inducer), and negative control (NAC) were assessed as a reference. A careful washing protocol was applied to remove any presence of DMSO before the addition of detection reagents to prevent interference. Based on the rapid decay of intracellular oxidant species, the quantification was acquired straight after the illumination protocol. As confirmed by all previous *in vitro* findings, the photosensitizer **3** was able to induce significant superoxide formation only when illuminated (Figure 4).

When the photosensitizer **3** was combined in the nanohybrids (**3/CNOs**), no significant deviation from the basal level of superoxide could be detected in HeLa cells, both prior to and after illumination, at each tested concentration. As a validation tool of the method applied, measurements related to pyocyanine exerted a considerable ROS level increase (ROS inducer), whereas NAC proves to decrease the ROS level. In order to confirm and combine the results with the cell metabolic activity protocol, a 24-h ROS quantification was determined (see Supplementary Material). While ROS could be detected immediately after illumination, no superoxide species could be detected 24 h after the illumination as a result of the massive cell death due to the phototriggered cytotoxic effects.

**Figure 5:**
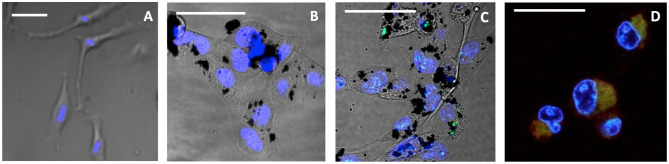
Confocal live images of HeLa incubated for 24 h, after 15-min illumination. **(A)** Untreated control, **(B)** treated with CNOs, **(C)** treated with **3/CNOs** at a mass concentration of 10 μg mL^−1^, **(D)** treated with **3**, at the corresponding concentration of 2.3 × 10^−7^ M. Nuclei were stained with Hoechst 33342 (blue), whereas green refers to the I_2_-BODIPY signal. Scale bars **(A–D)** = 50 μm. The presence of **3** or **3/CNO** is indicated by the green fluorescence signal, while intracellular oxidative damage is visible as red color (or yellow when overlapping with green). Scale bar = 50 μm.

To further confirm the obtained results, a confocal qualitative imaging analysis was conducted on live cells for intracellular superoxide species detection. HeLa cells previously treated for 24 h with the free photosensitizer **3** or **3/CNOs** were stained with Hoechst 33342 and CellROX® Deep Red fluorescent dye, before and after illumination, and observed (Figure 5).

When cells were exposed to **3** but kept in dark conditions (no illumination provided), cells appeared healthy and **3** was detected (Figure 2D), whereas after 15 min of illumination, the sample treated with **3** presented a remarkable red channel signal, indicating the interaction with CellROX® Deep Red fluorescent dye, which revealed cell oxidative damage (Figure 5D). Cells presented evident morphological changes, indicating death in the treated cells as a result of **3**'s phototoxic effects. As confirmed by all previous analysis, when **3** is coupled with **CNOs** (i.e., cells are treated with **3/CNOs**), no cell oxidative damage was evident, as no red signal related to superoxide species production was displayed. These results perfectly match the previously discussed experimental findings; thus, the CNOs are responsible for the low PDT efficiency of the nanohybrids due to the absence of ROS formation.

## Conclusions

In this study, a novel pyrene–diiodo-BODIPY dyad was synthesized, isolated, and characterized, revealing effective features as a photosensitizer. *In vitro* tests on HeLa demonstrated ROS-triggered phototoxicity of the photosensitizer **3**. The pyrene–diiodo-BODIPY dyad **3** was then successfully immobilized onto the surface of CNOs. Live-cell confocal imaging revealed that the pyrene–diiodo-BODIPY dyad **3** was effectively internalized by the cells, monitoring the residual fluorescence of the BODIPY chromophore. Nevertheless, illumination of the **3/CNO** nanohybrid-treated cells revealed no significant reduction in cell metabolic activity. These results indicated that the BODIPY photosensitizer **3** is deactivated when attached to the CNO surface. This observation may explain why, despite excessive research efforts in the field PDT as well as in the field of CNMs applied for cancer therapy, no relevant cytotoxicological studies are available. In this report, utilizing a non-covalent approach, we demonstrate for the first time that both the attachment of the photosensitizer to the CNM and the intracellular disruption of the delivery system to release the active agent are key pathways toward a successful PDT exploiting CNMs as molecular shuttles for drug delivery. The development of a feasible and efficient intracellular release function of the PDT agent will be on stage of future works in this field. Studies revealing the ROS generation features of the BODIPY photosensitizer in the presence and absence of CNOs matched these conclusions. Indeed, the present work elicits and broadens future research perspectives, which may involve the design “from scratch” of novel functional PDT-photosensitizer approaches and related apoptotic mechanisms toward the tumor.

## Data Availability Statement

All datasets presented in this study are included in the article/Supplementary Material.

## Author Contributions

JB conceived the idea, synthesized compounds, performed spectroscopic measurements, and wrote the manuscript. GM performed the biological experiments, analyzed the biological data, and revised the manuscript. VM performed biological experiments. Md'A performed imaging experiments and edited the manuscript. SB performed and analyzed mass spectroscopy data. SG conceived the idea, designed the experiments, supervised the chemical synthesis, the chemico-physical characterization and the biological experiments, and edited the manuscript. All authors contributed to the article and approved the submitted version.

## Conflict of Interest

The authors declare that the research was conducted in the absence of any commercial or financial relationships that could be construed as a potential conflict of interest.
