# Supplementary material for: Modulation of Efficient Diiodo-BODIPY in vitro Phototoxicity to Cancer Cells by Carbon Nano-Onions
Source: Front Chem. 2020 Oct 6;8:573211. doi: 10.3389/fchem.2020.573211 (PMC7574714; doi:10.3389/fchem.2020.573211)
Supplement: Supplementary file 1 [file Table_1.doc]

**Supporting information**

**Modulation of Efficient Diiodo-BODIPY in vitro Phototoxicity to Cancer Cells by Carbon Nano-Onions**

Juergen Bartelmess,^a^ Gesmi Milcovich,^a,b^ Viviana Maffeis,^a^ Marta d’Amora,^a^ Sine Mandrup Bertozzi ^a^ and Silvia Giordani^a,b^,*

^a^ Istituto Italiano di Tecnologia (IIT), Via Morego 30, Genoa, Italy

^b^ School of Chemical Sciences, Dublin City University (DCU), Dublin, Ireland

***Corresponding author:** Prof. Silvia Giordani. E-mail: silvia.giordani@dcu.ie

**Content:**

**Figure S1.** Characterization of compound **3**: FT-IR.

**Figure S2.** Characterization of compound **3**: ^1^H-NMR.

**Table S1.** Characterization of compound **3**: HRMS.

**Figure S3.** Characterization of compound **3**: LRMS.

**Figure S4.** Photobleaching characterization of **3**, **3/CNOs** and **3/benz-CNOs** upon illumination.

**Figure S5.** ROS quantification of **3/CNOs** 24h after illumination.

**Figure S6.** ROS quantification of **3/benz-CNOs**.

**Figure S7.** ROS quantification of **3/benz-CNOs** 24h after illumination.

**Characterization of compound 3:**


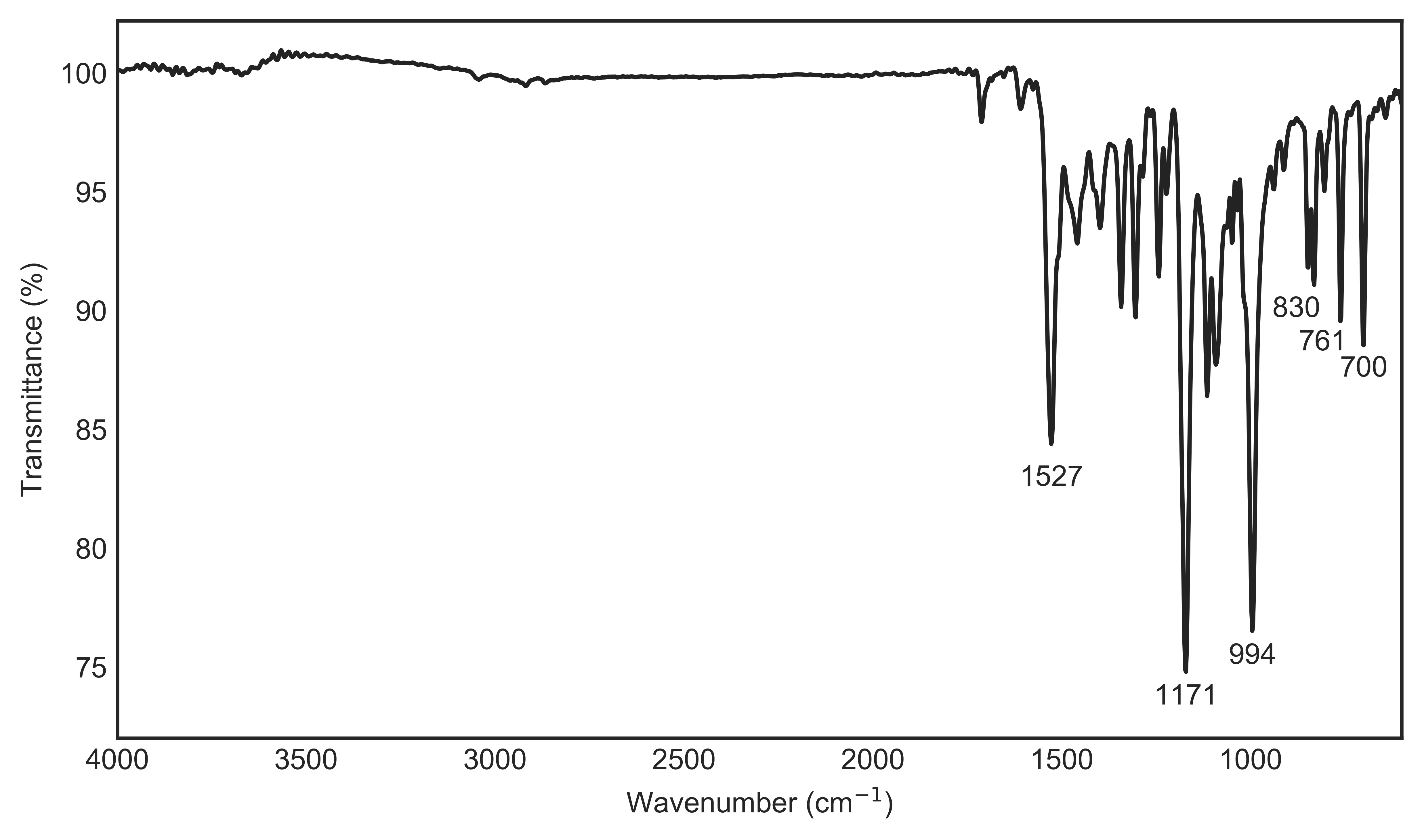


**Figure S1.** FT-IR spectrum of compound **3**.

**Figure S2.** ^1^H-NMR spectrum of compound **3**.

| **Formula** | **Monoisotopic Mass** | | **Measured Mass** | | **Calculated Mass** | | **Proposed Formula** | | | **Mass Accuracy (ppm)** | |
| --- | --- | --- | --- | --- | --- | --- | --- | --- | --- | --- | --- |
| C39 H30 B F2 I2 N5 O | 887,0601 | | 888,0691 | | 888,0686 | | C39 H31 B F2 I2 N5 O | | | 0,6 | |
|  | |  | |  | |  | |  |  | |  |

**Table S1.** HRMS data of compound **3**.


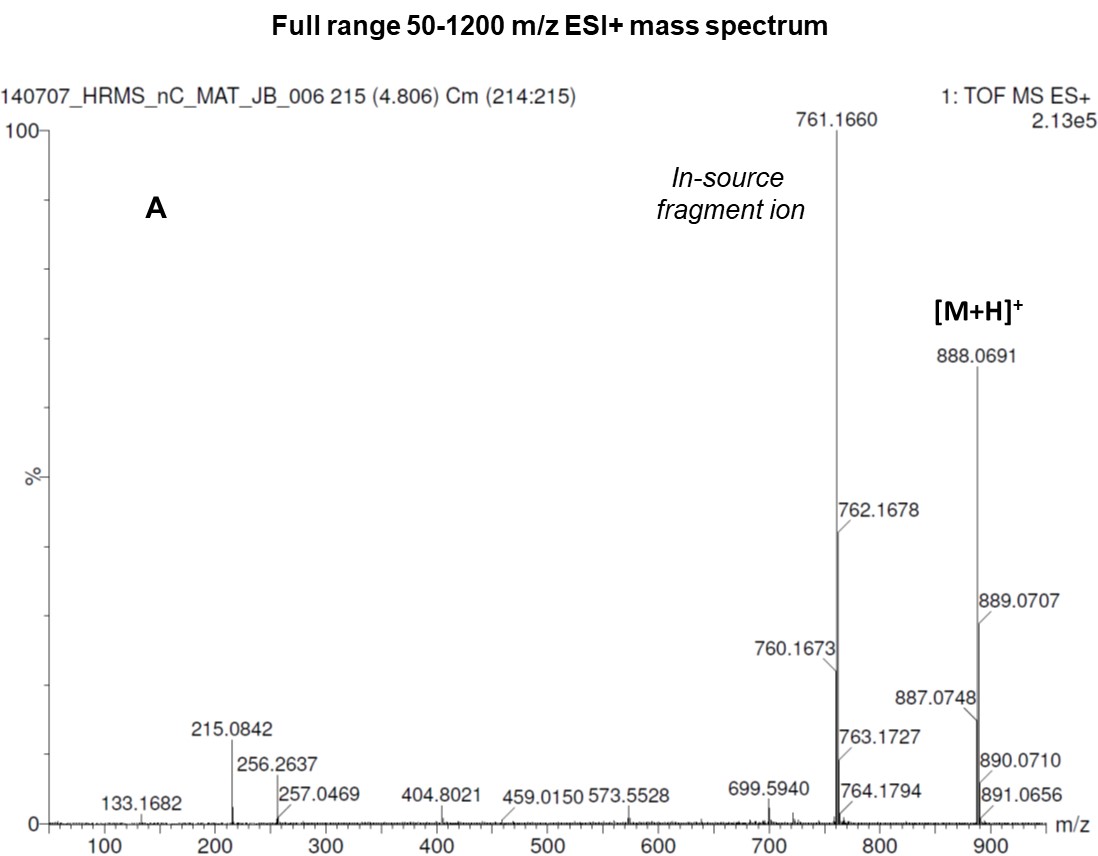


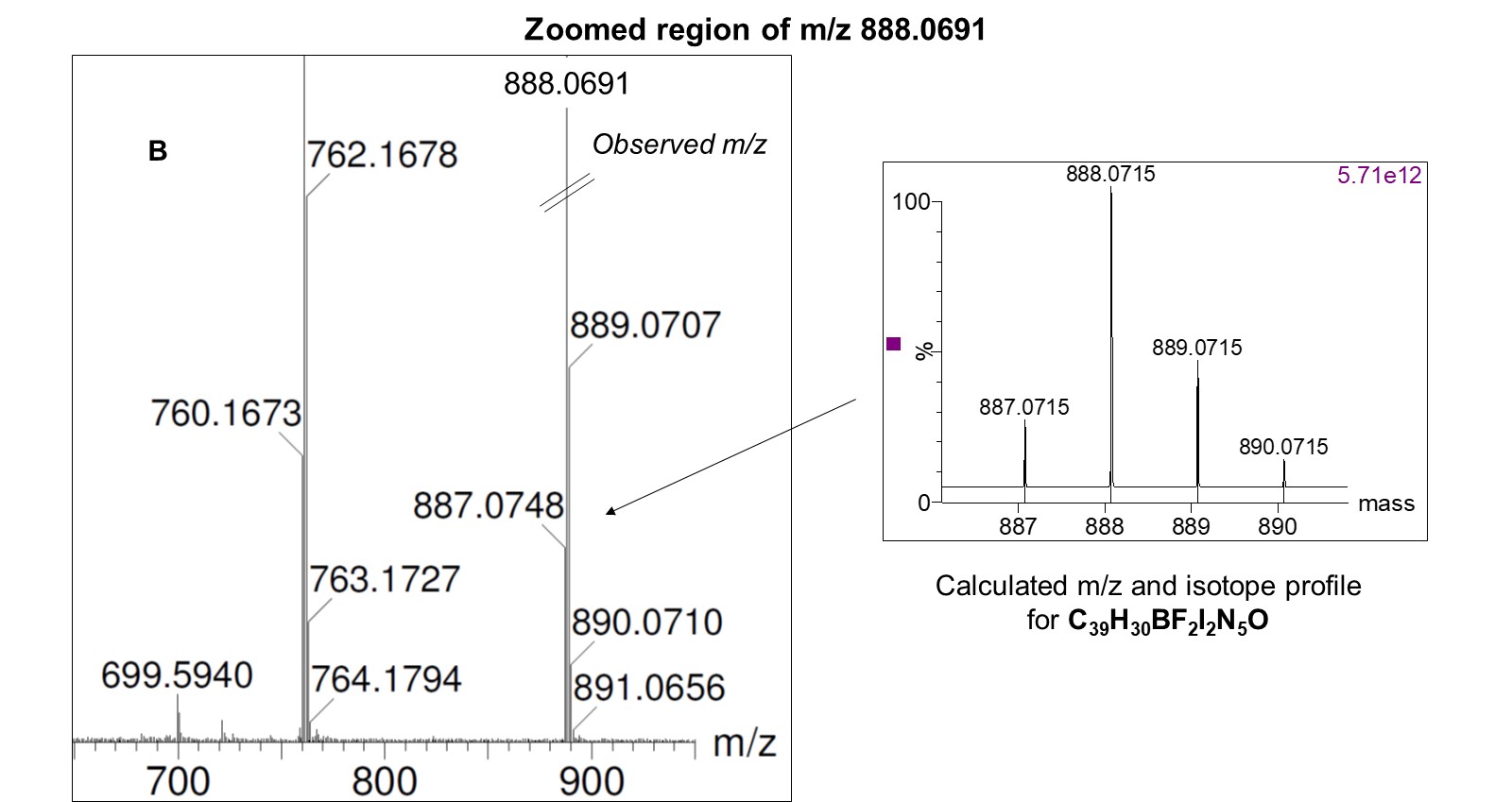


**Figure S3.** **A:** Full range m/z 50-1200 MS spectrum in ESI+ of compound **3**; **B:** Calculated m/z and isotopic profile of compound **3**.

**Photobleaching characterization of 3, 3/CNOs and 3/benz-CNOs upon illumination:**

**
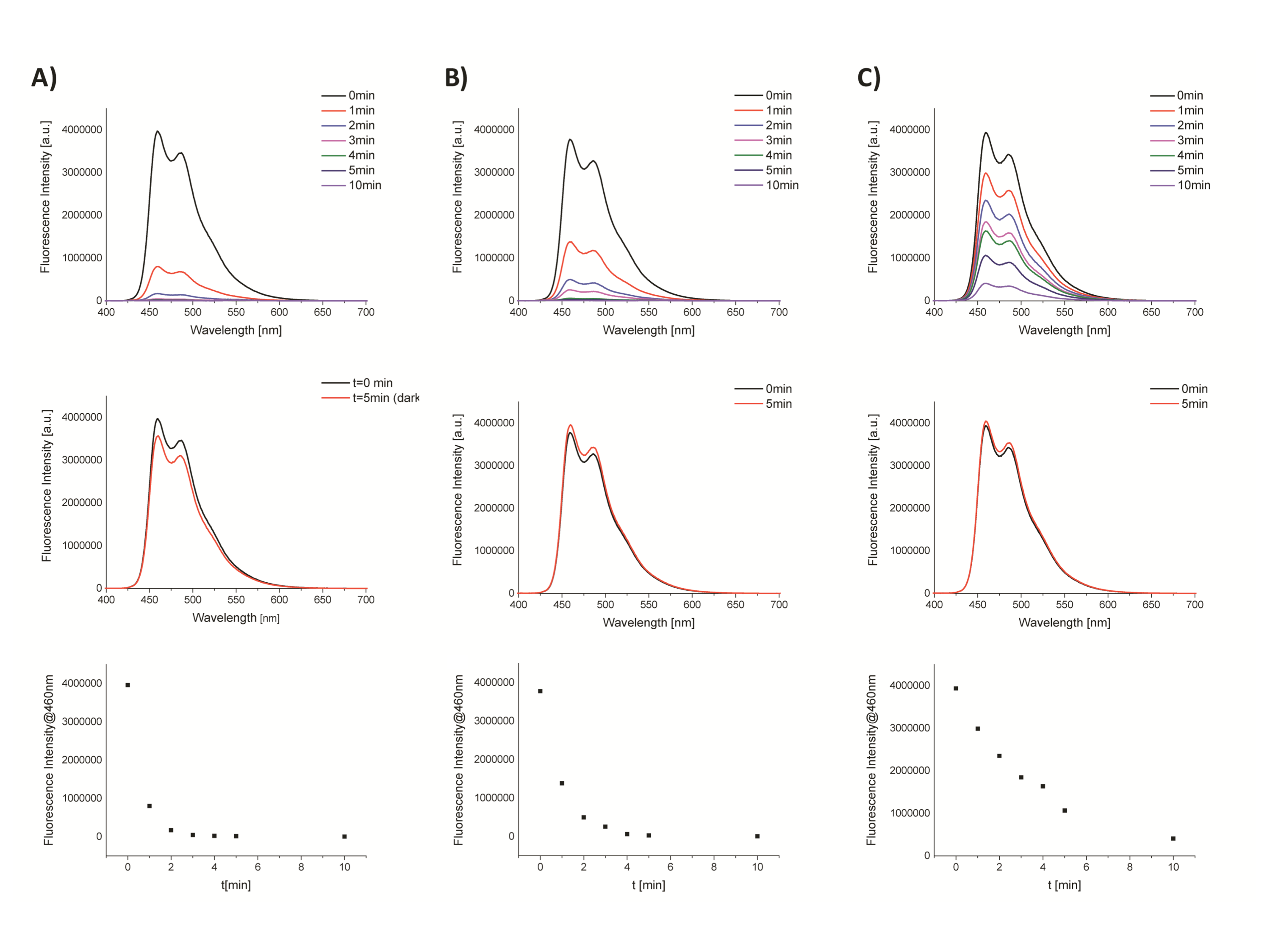
**

**Figure S4.** Fluorescence spectra of 1,3-diphenylisobenzofuran (DPBF) in the presence of **3** (A), **3/CNO s**(B) and **3/benz-CNOs** (C) upon 380 nm excitation in benzyl alcohol. Concentration DPBF 4.6 x 10^-6^ M. Emission spectra recorded at different time intervals between 0 and 10 min (top row) of illumination. Control experiments in the dark after 0 and 5 min (center row), and maximum fluorescence intensities as function of illumination time (bottom row).

**ROS quantification of 3/CNOs 24h after illumination:**

**
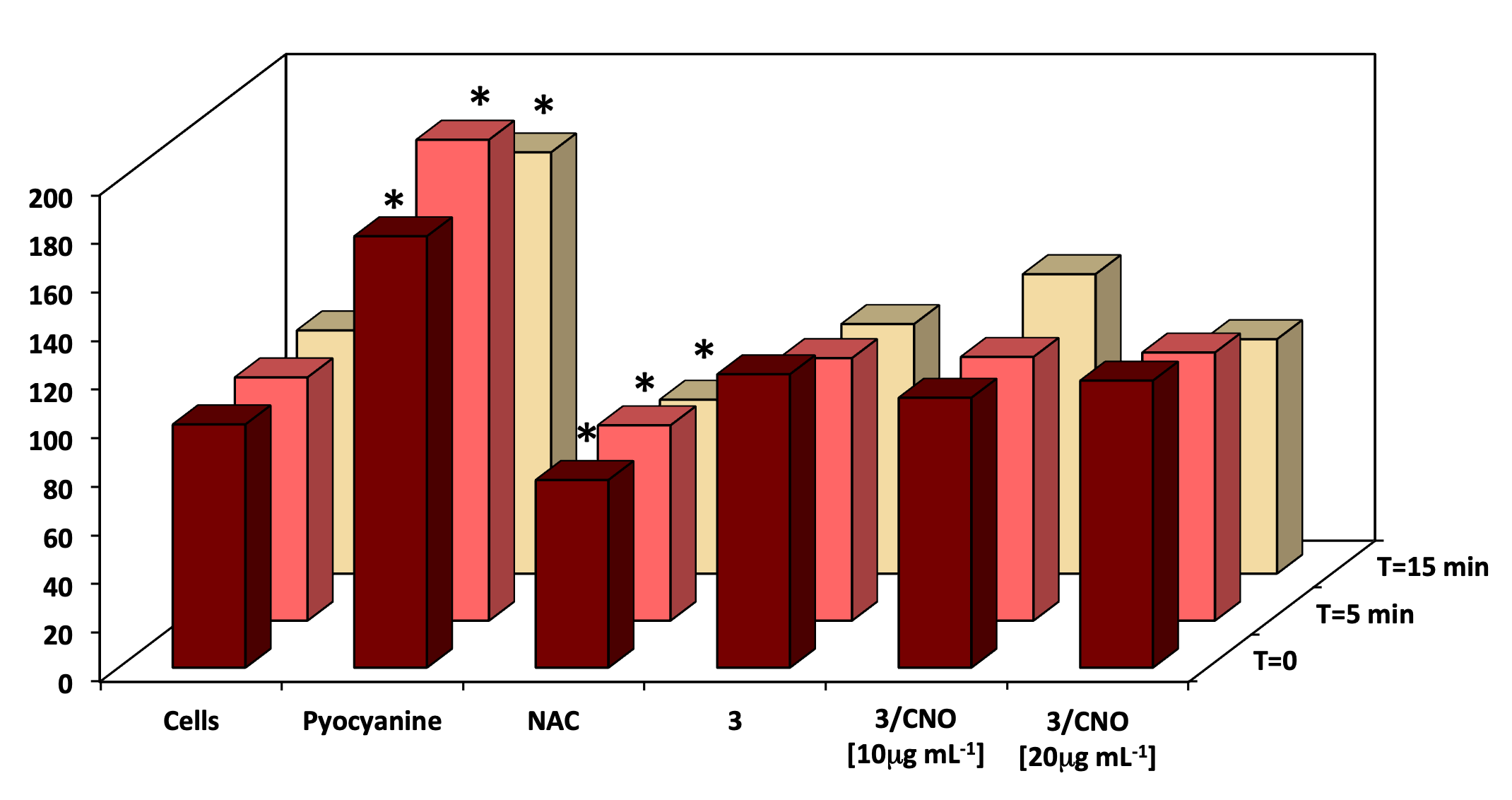
**

**Figure S5.** Superoxide level presence and quantification in HeLa cells incubated with different mass concentrations of **3**, **3/CNOs** and following illumination at different time points (n=3, p<0.05). Basal control (cells), positive control (pyocyanine, ROS inducer) and negative control (NAC, N-acetyl-L-cysteine) were provided.

**ROS quantification of 3/benz-CNOs:**

**
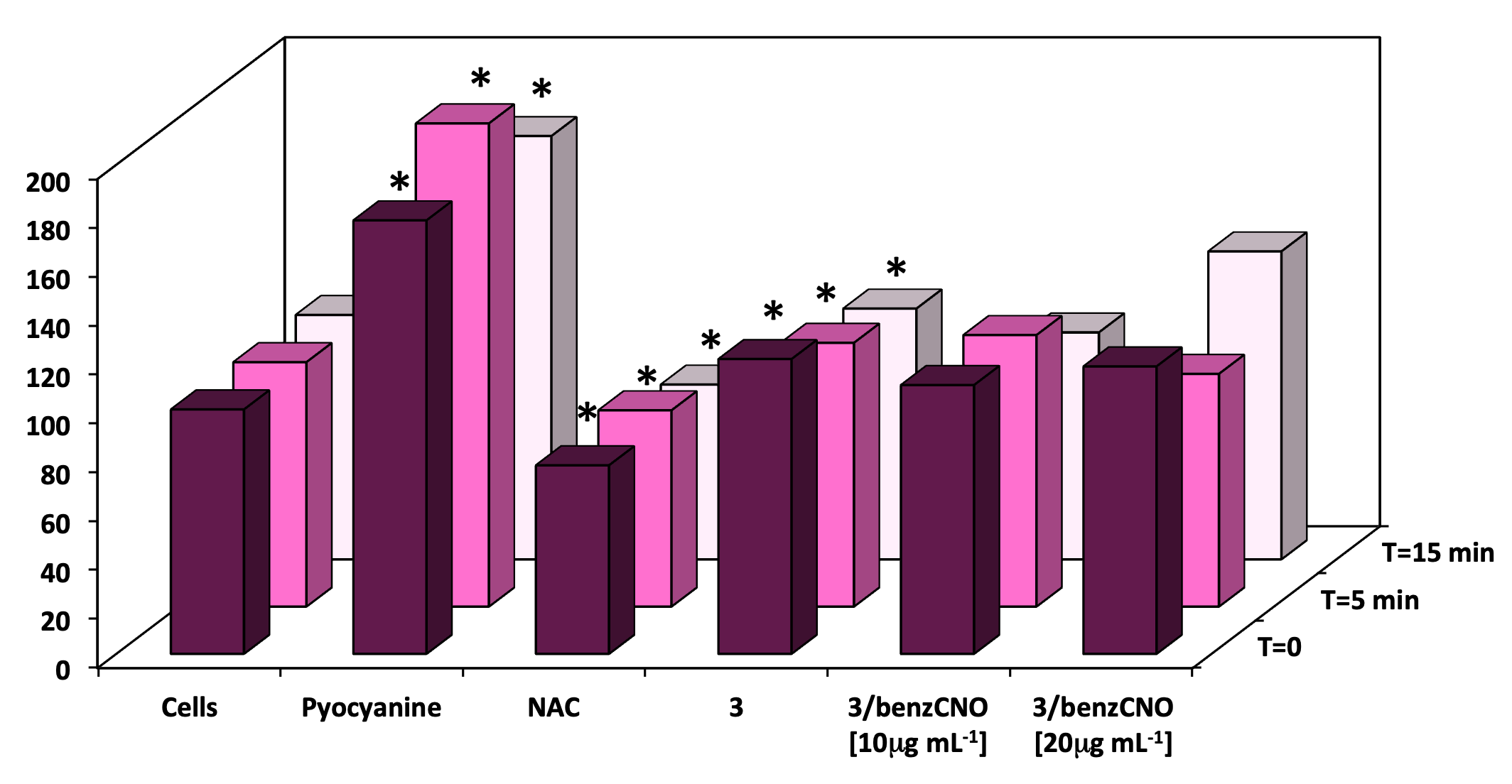
**

**Figure S6.** Superoxide level presence and quantification in HeLa cells incubated with different mass concentrations of **3** or **3/benz-CNOs** and following illumination at different time points (n=3, p<0.05). Basal control (cells), positive control (pyocyanine, ROS inducer) and negative control (NAC, N-acetyl-L-cysteine) were provided.

**ROS quantification of 3/benz-CNOs 24h after illumination:**

**
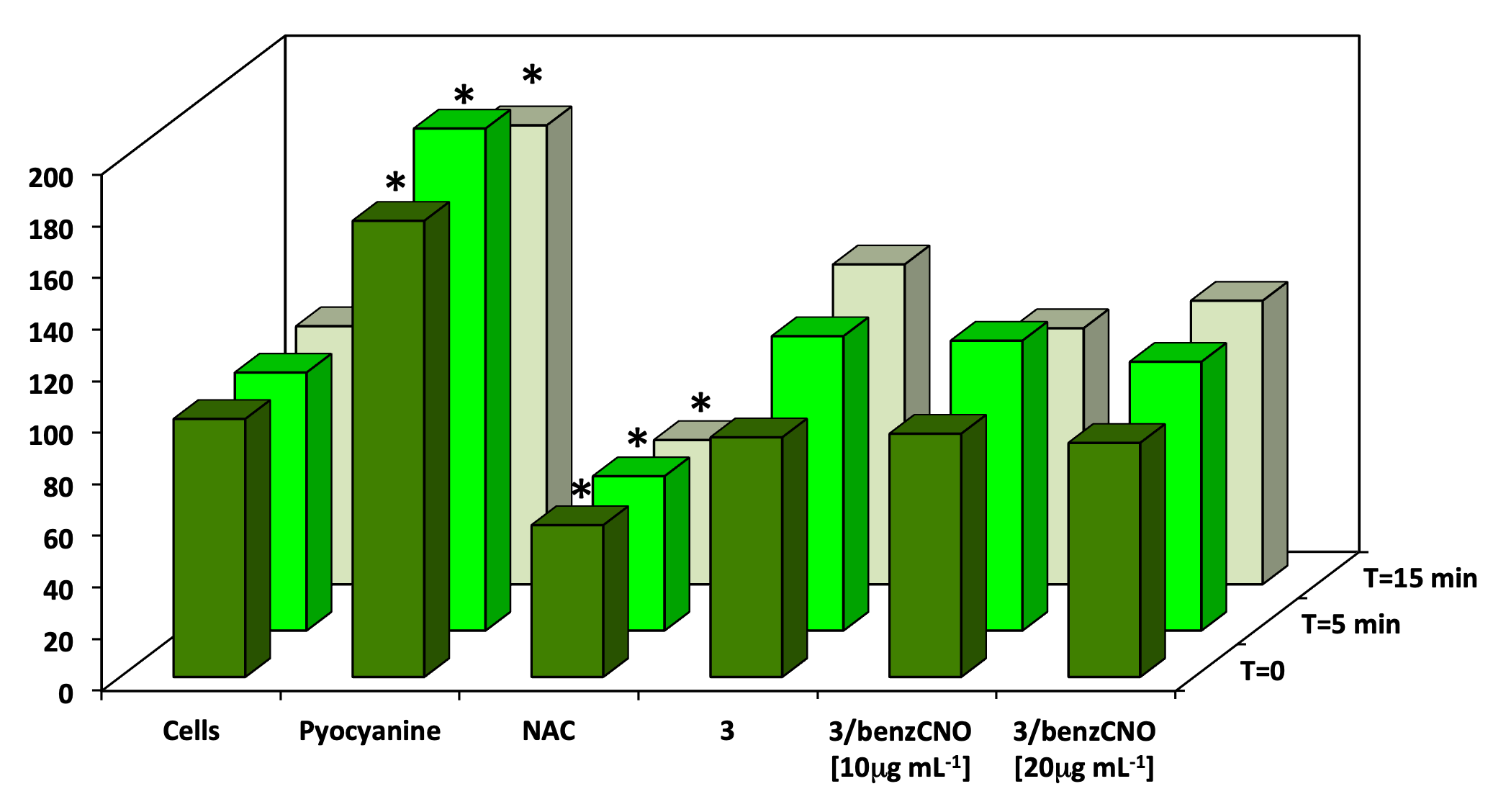
**

**Figure S7.** Superoxide level presence and quantification in HeLa cells incubated with different mass concentrations of **3 or** **3/benz-CNOs** and following illumination at different time points (n=3, p<0.05). Basal control (cells), positive control (pyocyanine, ROS inducer) and negative control (NAC, N-acetyl-L-cysteine) were provided.
